# The Use of HPTLC and SDS-PAGE Methods for Coniferous Honeydew Honey Fingerprinting Compiled with Mineral Content and Antioxidant Activity

**DOI:** 10.3390/molecules27030720

**Published:** 2022-01-22

**Authors:** Monika Tomczyk, Aleksandra Bocian, Ewelina Sidor, Michał Miłek, Grzegorz Zaguła, Małgorzata Dżugan

**Affiliations:** 1 Department of Chemistry and Food Toxicology, Institute of Food Technology and Nutrition, College of Natural Sciences, University of Rzeszow, 35–601 Rzeszow, Poland; bocian@prz.edu.pl (A.B.); ewelina.sidor.dokt@gmail.com (E.S.); mmilek@ur.edu.pl (M.M.); mdzugan@ur.edu.pl (M.D.); 2 Department of Biotechnology and Bioinformatics, Faculty of Chemistry, Rzeszow University of Technology, 35–959 Rzeszow, Poland; 3 Doctoral School, University of Rzeszow, Poland, 35–959 Rzeszow, Poland; 4 Department of Bioenergetics, Food Analysis and Microbiology, Institute of Food Technology and Nutrition, College of Natural Sciences, University of Rzeszow, 35–601 Rzeszow, Poland; gzagula@ur.edu.pl

**Keywords:** honeydew honey, sugars, polyphenols, protein, antioxidant activity, authenticity, HPTLC, SDS-PAGE

## Abstract

Fir honeydew honey is a uniquely beneficial product which is often subjected to adulteration; however, pollen analysis is not useful to verify this honey type. Fourteen samples of EU protected designation of origin fir honeydew honey gathered directly from apiaries were studied. Standards of legal requirements and additional parameters, i.e., specific optical rotation, mineral content, and antioxidant activity, were tested. Five nectar honeys of different varieties were used as a comparative material. HPTLC and SDS-PAGE methods were used to fingerprint the honey types. All honeys tested fulfilled the quality requirements in terms of water content, pH, total acidity, conductivity, HMF, and diastase number. They were defined as dark amber on the Pfund scale and exhibited positive specific rotation (+2.5 to 25). Honeydew honey surpassed the tested nectar honeys in terms of mineral content and antioxidant activity as well as total polyphenolic content, except for buckwheat honey. The sugar and polyphenolic profile obtained by HPTLC allowed to distinguish honeydew from nectar honeys. The same was achieved by SDS-PAGE protein profiling. Both techniques seem to be cheap and quick tools for precisely distinguishing honeydew honey.

## 1. Introduction

Honeydew honey is produced by bees (*Apis mellifera*) from secretions from living parts of plants or excretions of plant-sucking insects (*Hemiptera*) such as aphids and scale insects on the living part of plants [1]. The plants used for honeydew honey production are mostly coniferous trees such as fir (*Abies alba*), and spruce (*Picea abies*), but also leafy trees, such as oak (*Quercus* spp.) and lime (*Tilia* spp.) [2]. When insects are fed plant sap, they absorb essential nutrients and secrete the sugar-rich sticky liquid remains known as honeydew. These exudates are collected by honeybees and turned by them into honey known as honeydew-, forest-, fir- or spruce-honey [3]. Therefore, the components of honeydew honey originate from phloem sap (botanical origin), from the honeydew producing insect (zoological origin), and are added by bees in the process of honey production (additional zoological origin) [3]. The composition of honeydew varies between different species of sap-sucking insects including aphids, leafhoppers, and psyllids, and is also influenced by the host plant species and different environmental conditions [4]. 

The chemical composition as well as the biochemical and physiochemical parameters of honeydew honey differ from those of flower honey (based on floral nectar). Honeydew honey generally exhibits higher values of electrical conductivity, pH, ash percentage, higher content of disaccharides and trisaccharides, and lower level of monosaccharides. It is characterized by a darker color and peculiar sensory features compared to blossom honeys [5,6,7,8]. Due to the specific origin of honeydew honey, its market price is higher than that of nectar honeys. As a result of this, honeydew honey is often subject to adulteration with nectar honey. Actually, honeydew honey is labeled on the basis of sensory judgements and electrical conductivity values (the minimum required is 0.8 mS/cm), but there is no internationally accepted quality criterion for different types of honeydew honeys [9]. For flower honey, pollen analysis (melissopalynology) is used to identify the botanical origin; this method, however, is not useful for honeydew honey [7]. Scientists have attempted to distinguish honeydew from nectar honeys on the basis of their mineral composition, physicochemical properties, the presence of characteristic flavonoids, sugar profile, enzymatic and antioxidant activity, but so far, no easy and quick technique has been developed to clearly distinguish honeydew from nectar honey [8].

Fir honeydew honey is a typical product of forest beekeeping in the Podkarpacie region (southeastern Poland), which is rich in coniferous forests, abundant in the European silver fir (*Abies alba*). This top-quality product has been on the EU’s protected designation of origin list since August 2010 [10]. This honey has a special, spicy flavor and delicate, sweetish, typically resinous aroma as well as a unique dark brown color with greenish reflections. The special qualities of Podkarpacie honeydew honey result from the ecologically clean natural environment (the region includes two national parks and 15 nature reserves), specific soil conditions, and the elevation of the terrain favorable the growth of coniferous trees. Local beekeepers who collect honey in the traditional way dating back centuries are greatly skilled and committed to preserving traditional apiary management closely connected with this region. They know how to accurately choose the location of the apiary in view of the amount of honeydew available, gather the unique honey, and properly store and package it [11].

The biological activity of Podkarpacie honeydew honey has not been studied in detail until now. However, various honeys have been used for centuries as a traditional medicine in different cultures, showing specific properties in the treatment of various disorders. The healing properties of honey are related to its antioxidant, antimicrobial, anti-inflammatory properties [12,13,14,15,16,17]. Due to the fact that honeydew, honey usually presents higher contents of bioactive compounds such as phenolics, proteins, amino acids, and enzymes as compared to blossom honeys, it also exhibits higher antioxidant and antimicrobial activities [8,15], indicating that this type of honey is potentially extremely health-promoting. Honeydew honey has been investigated through in vivo and in vitro studies as a promising agent in the treatment of wounds [18], non-healing leg ulcers [19], eye wounds [20], skin disorders [21] and the gastrointestinal system [22]. 

In this study, the polyphenolic and protein profile, physicochemical properties, and antioxidant activity of the EU’s protected designation of origin fir honeydew honey gathered directly from apiaries were studied in detail. Moreover, simple fingerprinting with HPTLC and SDS-PAGE methods, highly anticipated by the beekeeping industry, was provided.

## 2. Results and Discussion

### 2.1. Physicochemical Parameters

The EU Directive from 2014 [1] clearly indicates the quality requirements for varietal honeys. According to its guidelines, varietal honeys cannot contain more than 20% water, except for heather honey, where the value of this parameter cannot exceed 23%. According to the results presented in this study, honeydew honey is characterized by a relatively low water content, which was on average 16.94%, but did not exceed 19.10% (Table 1). A similarly low level of water content in Polish honeydew honeys was found by Madejczyk and Barałkiewicz [23] and Rybak-Chmielewska et al. [24], where moisture was less than 20% in all honey samples. Additionally, in European honeydew honeys, Persano Oddo and Piro [25] determined that average water content was 16.1%. The water content in Slovenian honeydew honeys ranged from 13.4% to 18.0% [26]. Moreover, the results indicated that there were no statistically significant differences (*p* > 0.05) between honeydew honeys and other varieties tested.

The mean pH value of this tested bee product was 4.66 and was similar to the results for honeydew honeys presented by other authors: 4.63 [24], 4.60 [8] and 4.48 [27]. Honeydew honey is known to show higher average acidity and pH than floral honeys [6], which was also proven in the present study. The results obtained indicate that the average acidity of honeydew honeys was in the range 37.70–65.50 mval/kg, with the average value of 48.20 mval/kg. Slightly lower values of Polish honeydew honeys were obtained by Kaczmarek et al. [28]. However, European honeys analyzed by Balos et al. [29] showed much lower average acidity values (32.67 mval/kg). It is commonly accepted that honey acidity changes during storage and depends on geographical origin; however, our results indicate that it is also related to honey variety. The acidity of honeydew honey acidity, measured in this study, was significantly higher as compared to acacia, multifloral and rape honeys (*p* < 0.05).

As the EU Directive [1] clearly indicates, to classify honey as honeydew, its conductivity must be greater than 0.8 mS/cm. This parameter can be helpful in identifying honeydew honeys. For other honey varieties, the electrical conductivity must be in the range from 0.2 to 0.8 mS/cm. In the analyzed honeydew honeys, the average value of electrical conductivity ranged from 1.028 to 1.414 mS/cm, which clearly indicates that the honeys can be classified as honeydew. The results obtained are comparable with the values presented by other authors: 1.140 [24] or 1.10 mS/cm [8]. Slightly lower values for dark honeys were obtained by Madejczyk and Barałkiewicz [23]; however, the value of 0.9 mS/cm was within the required parameters for honeydew honeys [1]. The present study also proved that conductivity could be used as a parameter to distinguish honeydew honeys from nectar honeys where this parameter was significantly higher (*p* < 0.05) than in nectar honeys tested.

HMF and diastase activity serve as the markers of honey overheating and it is covered by commercial requirements and should be less than40 mg/kg. In all samples tested, HMF content was well below the maximum level. It is known that the HMF level increases during the thermal treatment of honeydew honey [30]. As lower values were obtained for fresh honeys than for stored ones, the formation of HMF during prolonged storage of this type of honey can be its specific feature. No such strong differences were found in activity of α-amylase determined by the diastase number (DN); however, the mean value for fresh honey was 30–40% higher than for stored sample honey, which is in agreement with our earlier findings that during prolonged storage (24 months) at room temperature, the diastase activity decreased by 30% [30]. The values of this parameter were relatively low compared to reported literature data for honeydew honeys from Poland with an average value of 28.4 [24]. However, the average value of 13.20 and even the lowest value of 8.92 fulfilled legal demands.

The acceptability of honey by the consumer and its market price depend mainly on the color of the honey. According to the classification based on Pfund mm scale set by USDA [31], color of honey ranges from water white (<8 mm) to dark amber (>114 mm). In the EU, this parameter is not evaluated during quality assessment. Based on the Pfund classification, all tested honeydew honeys were classified as dark amber. The color of honey was reported to be affected by the chemical composition, primarily the presence of pigments such as chlorophylls, carotenoids, flavonoids, and polyphenols [32,33]. Honeys with dark color are generally known for their antioxidant activity and their high ash content, while those with light color in the opposite show much lower values of the mentioned parameters [33,34]. In the present study, significant differences in color were found between honeydew honeys and other tested nectar honeys (*p* < 0.05) except buckwheat (*p* > 0.05).

Specific rotation is one of the physicochemical parameters that clearly distinguishes honeydew honeys from nectar honeys, showing a positive and negative value of this parameter, respectively [5,7]. All tested honeydew honeys showed positive rotation, albeit diversified in the range +2.5 to +25, which confirms their honeydew origin. In contrast, the compared nectar honeys showed negative specific rotation, from −17.92 (buckwheat) to −43.33 (acacia). These results find a confirmation in the literature: for Bulgarian honeys, the specific rotation was determined at the level of −17 for acacia, −14.8 for multifloral and +4.2 for honeydew [35]. Tarapatskyy et al. [27] obtained on average +2.85 for Podkarpackie honeydew honey, while for other varieties they tested a negative value.

### 2.2. Mineral Composition

The concentrations of the elements tested in the honeydew honey samples are shown in Table 2. Among the microelements analyzed (Sr, Zn, Cu, Fe, Mn), the highest variability for Zn (VC 134%) was found, which ranged from 0.66 to 9.20 mg/kg. The microelement with the most stable concentration in the samples was Cu (VC 12.6%) and the mean value for all samples was 1.15 mg/kg. The most abundant macroelement in all tested samples was K, with a mean value of 2572.64 mg/kg. Moreover, its concentration was not significantly diversified (VC 10%) in particular samples. The most diverse macroelements in terms of variability between individual samples was Ca (VC 70%), with a concentration ranging between 11.21 and 115.78 mg/kg. The results obtained are strongly supported by other authors’ findings [36]. Madejczyk and Baralkiewicz [23] studied the mineral content of honeydew honey originating from various Polish locations, including samples from the Podkarpacie region. They also showed that K is the most abundant mineral, with a mean concentration of 2612.2 mg/kg. It was tested earlier that the concentration of elements in honeydew honey (dark honey) is several times higher than in pale nectar honeys [7]. In the study of Terrab et al. [37], honeydew honeys were perfectly distinguished from other honeys tested by a very high content of minerals and electrical conductivity. In the study cited, the content of minerals allowed for a varietal differentiation of only honeydew honeys (100% correct classification). In the honeys analyzed, K was also the dominant element (80% of all quantified minerals). 

The analysis of toxic elements shows that the content of Cd ranged between 0.02 and 0.05 mg/kg, while Pb 0.02 and 0.12 mg/kg. In current legislation, the concentration of Cd in honey is not limited and the maximum level of Pb should not exceed 0.1 mg/kg [38]. In our tests, only two honeydew honey samples exceeded this level. It was previously proven that Podkarpackie honeydew honey exhibit increased values for Cd compared to other countries, which is caused by the specificity of the soil in this region [39,40]. The specific high content of Al in honeydew honey was previously found in Polish samples by other authors [23,36,40] and is related to the honeydew susceptibility to contamination with this element. Of all the toxic elements tested, only As did not show significant differences (*p* > 0.05) between samples. 

The total content of elements in particular samples ranged between 0.30 and 0.37% and is lower than expected. According to literature data, the total mineral content in honey generally accounts for 0.1% to 0.2% of the composition of nectar honeys, but it can exceed 1% in other types of honey, such as honeydew honey [41]. However, the mineral content of honey is the parameter that strongly depends on geographical origin [23,36,37,40,41].

### 2.3. Antioxidant Activity

The total content of phenolic compounds in the tested honeydew honey samples ranged from 635.42 to 1289.43 mg GAE/kg (Table 3). According to the literature data, honeydew honey is one of the richest in terms of polyphenol content. Values similar to those obtained in this study were observed for Romanian (935–1449.4 mg GAE/kg g) and Bosnian honeys (1361–1570 mg GAE/kg) [42,43]. According to other studies, Polish honeydew honeys contained from 582.4 to 718.8 mg GAE/kg, and in terms of TPC they were weaker than samples of only buckwheat honey [44,45], which was also confirmed in the present study. The antioxidant capacity values tested by three different methods indicate that honeydew honey is a rich source of antiradical and reducing agents. In terms of FRAP reducing ability, the tested honeydew honeys are close to the average tested in our previous studies for honeydew honeys from this region (533.30 mg TE/kg vs. 538.12 mg TE/kg for coniferous honeydew honeys, 505.59 mg TE/kg for leafy coniferous honeys and 710.82 mg TE/kg for forest honeys) [15,46]. The antiradical activity is also comparable to the literature data. Goślński et al. [47] determined the DPPH radical scavenging capacity at the level of 416 mg TE/kg, which was the highest result among the samples tested, including Manuka honey [47]. For forest honeys from central Serbia, the scavenging activity was 260.77 and 594 mg TE/kg, for DPPH and ABTS methods, respectively [48]. Moreover, our results indicate that the antioxidant activity of honeydew honey measured by different methods was significantly higher than in tested nectar honeys (*p* < 0.05) except for buckwheat honey (*p* > 0.05).

### 2.4. HPTLC Fingerprinting

#### 2.4.1. Sugar Analysis

**Figure 1:**
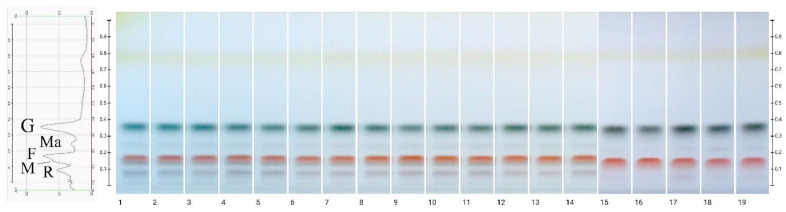
Images of HPTLC plates taken after derivatization by aniline—diphenylamine—phosphoric acid reagent in visible light, presenting sugars separation. Tracks 1–14—honeydew honeys, 15—heather, 16—acacia, 17—multifloral, 18—buckwheat, 19—rape honeys. (R—raffinose, M—melezitose, F—fructose, G—glucose, Ma—Maltose).

Fourteen honeydew honeys were analyzed for their HPTLC sugar profiles (Figure 1) compared to five different nectar honeys (heather, acacia, multifloral, buckwheat, and rape). The following bands were determined: glucose as a green band (Rf = 0.35), sucrose as a brown band (Rf = 0.28), maltose as a blue band (Rf = 0.22), fructose as an orange band (Rf = 0.14), melezitose as a brown band (Rf = 0.12) and raffinose as a brown band (Rf = 0.08). In all honey samples tested, a comparable content of glucose and fructose was determined. Maltose was found in all samples only in traces whereas saccharose was not detected. According to Manzanares et al. [6] maltose is a disaccharide present in Spanish honeydew in high amounts (about 5.5 g/100 g). The sugar profiles obtained were similar for all honeydew honey samples. Moreover, results have shown that raffinose and melezitose were specific for only honeydew honeys (they were present in mutlifloral honey only as traces). This means that the presence of melezitose and raffinose could be the marker of honeydew honey, which was proposed previously by other authors [3,27,49] by the HPLC method, but was not proved before using the HPTLC technique.

Because fructose and glucose are the major constituents of honey, their identification and quantification are of great interest for honey researchers. In particular, the fructose to glucose ratio (F/G) is an important parameter for the beekeeping industry, as it corresponds to the authenticity of the botanical origin of honey, helps in the identification of honey adulteration (e.g., feeding bees with sugar syrups during periods of low nectarization or adding sugar syrups to honey to increase honey production efficiency [50,51,52] and also allows to predict the form and speed of honey crystallization [30,50,53]. In the present study, the F/G ratio calculated based on fructose and glucose peak high from the HPTLC chromatograms in honeydew honeys ranged between 0.75 and 0.94, while for other honey types tested, except acacia honey (0.78), it varied from 0.64 to 0.70. The F/G ratio calculated by Islam [50] based on the same technique was significantly higher as compared to the present study and reached 1.74 for multifloral and 1.50 and 1.79 for unspecified honeys. Similarly, the F/G ratio calculated based on the HPLC method indicates higher values than in present study [6,27,49,54,55]. Such discrepancies are probably because during the HPTLC separation, it is impossible to split fructose from melezitose peaks and as a consequence a broad, irregular fructose peak with a subtle melezitose hump is not exactly delineated. To conclude, the HPTLC technique allows a qualitative analysis of the sugars in honey, but the quantitative analysis is not reliable.

#### 2.4.2. Polyphenolic Profile

**Figure 2:**
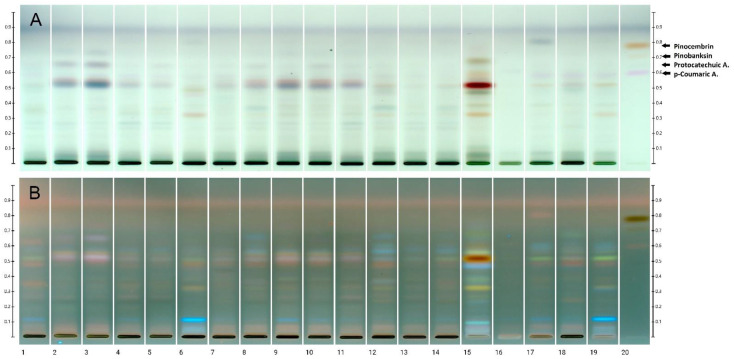
Images of HPTLC separation of honeydew (tracks 1–14) and nectar honeys (track 15—heather, 16—acacia, 17—multifloral, 18—buckwheat, 19—rape) after p-anisaldehyde derivatization, in UV 366 nm light (**A**) and visible light (**B**). Track 20 standards (in order of increasing Rf values): p-coumaric acid, protocatechuic acid, pinobanksin, pinocembrin.

The polyphenol profiles obtained by the HPTLC technique for honeydew honey presented 13 dominant bands, and only part of them were specific for this kind of honey, whereas others occurred also in the polyphenolic profiles of nectar honeys. Three of them (Rf 0.121, 0.576, and 0.646) are present in all honeydew honeys tested but only one of them (Rf = 0.646) is specific for honeydew honey (Table 4). However, based on its color after derivatization with p-anisaldehyde we suppose a different nature of this metabolite, more likely a terpene rather than a phenolic compound. Furthermore, the band at Rf = 0.563 is present in most honeydew honey samples but is not observed in any other nectar honey. The band with Rf = 0.524 was assigned to p-coumaric acid, which is commonly present in all honey varieties, including honeydew [56,57,58]. A repeated sequence of bands has been found to be characteristic of honeydew honey (Figure 2). Among the samples tested, the numbers 2, 3, 4, 5, 8, 9, 10, and 11 match it. Samples 1, 6 and 12 clearly stand out from the averaged fingerprint; they can be considered as having an admixture of other honeys or only nectar or pollen, e.g., rapeseed. Attempts have been made to use this type of fingerprints to determine the authenticity of varietal, goldenrod, and phacelia honeys, as well as to distinguish between particular varieties [59,60,61]. The most frequently recurrent polyphenols determined in honeydew honeys were protocatechuic, p- hydroxybenzoic acid, caffeic acid, p-coumaric acid, ferulic acid and abscisic acid, as well as flavonoids: chrysin and pinocembrin [57,58,62]. According to the literature data, protocatechuic acid (3,4-dihydroxybenzoic acid) is commonly present in all honeydew and forest honeys. This compound was proposed as a marker that distinguishes honeydew honeys from nectar honeys [62]. However, the analysis did not detect this compound in the samples tested.

**Figure 3:**
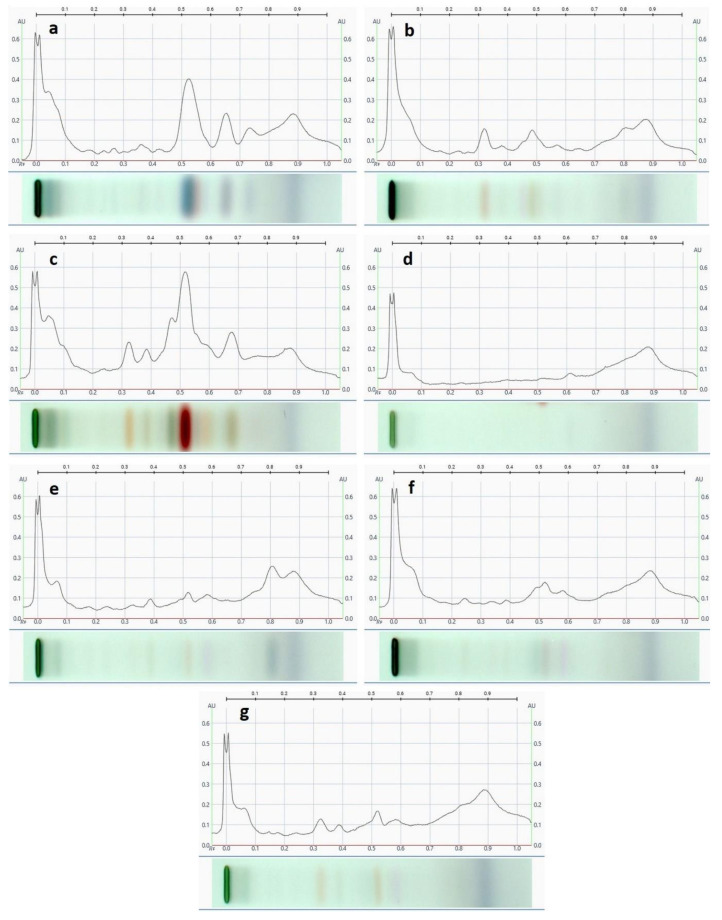
HPTLC polyphenolic profile to distinguish fir honeydew honey tested samples (**a,b**) and other nectar honeys: heather (**c**), acacia (**d**), multifloral (**e**), buckwheat (**f**), and rape (**g**).

Based on the comparison of the polyphenol profiles of honeydew honeys, two types of fingerprint can be distinguished (Figure 3). The first is a typical profile for fir honeydew honey (Figure 3a), which includes samples no. 2, 3, 4, 5, 7, 8, 9, 10, and 11. The second type, which is similar to the profile of nectar honeys (Figure 3b), in which the remaining samples can be included, indicates an admixture of nectar. For better visualization polyphenol profiles of nectar honeys have been presented (Figure 3c–g).

### 2.5. SDS-PAGE Fingerprinting

**Figure 4:**
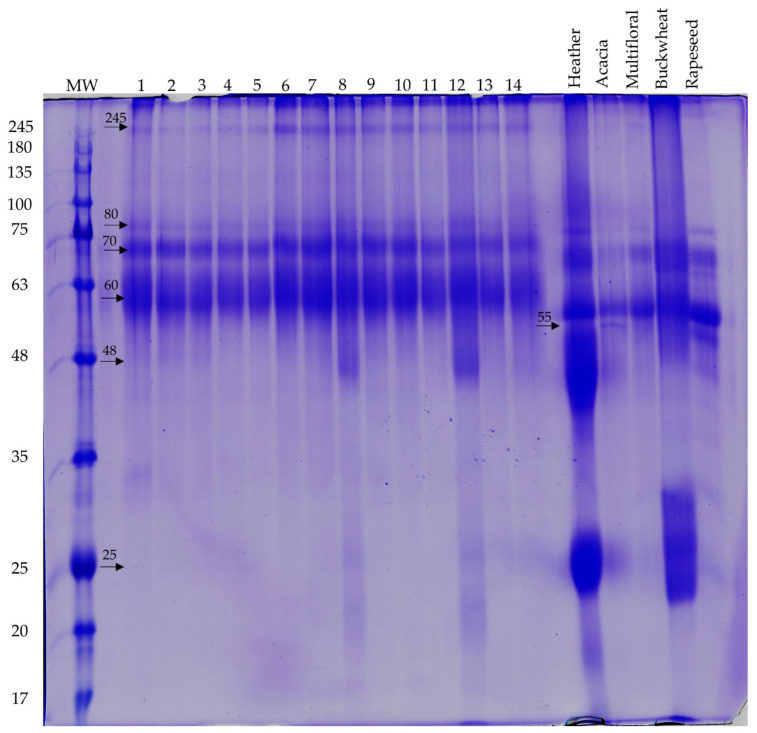
Qualitative SDS-PAGE gel stained with CBB-R. Arrows indicate the observed bands.

Protein profiles obtained using SDS-PAGE electrophoresis were similar for all analyzed honeydew honeys and completely different in the range of high and low molecular weight proteins compared to nectar honey profiles. The similarity of protein profile is particularly evident for the three bands (80, 70, and 60 kDa) that are seen in all the analyzed honey regardless of its variety. However, while the 70 and 80 kDa bands look similar in all samples, the lowest one at 60 kDa is more blurred and wider in all honeydew honey samples than nectar honeys (Figure 4). The slight differences in the migration rates of the major protein fractions between honeydew and nectar honeys are probably due to the content of some interfering components of the honey samples.

**Figure 5:**
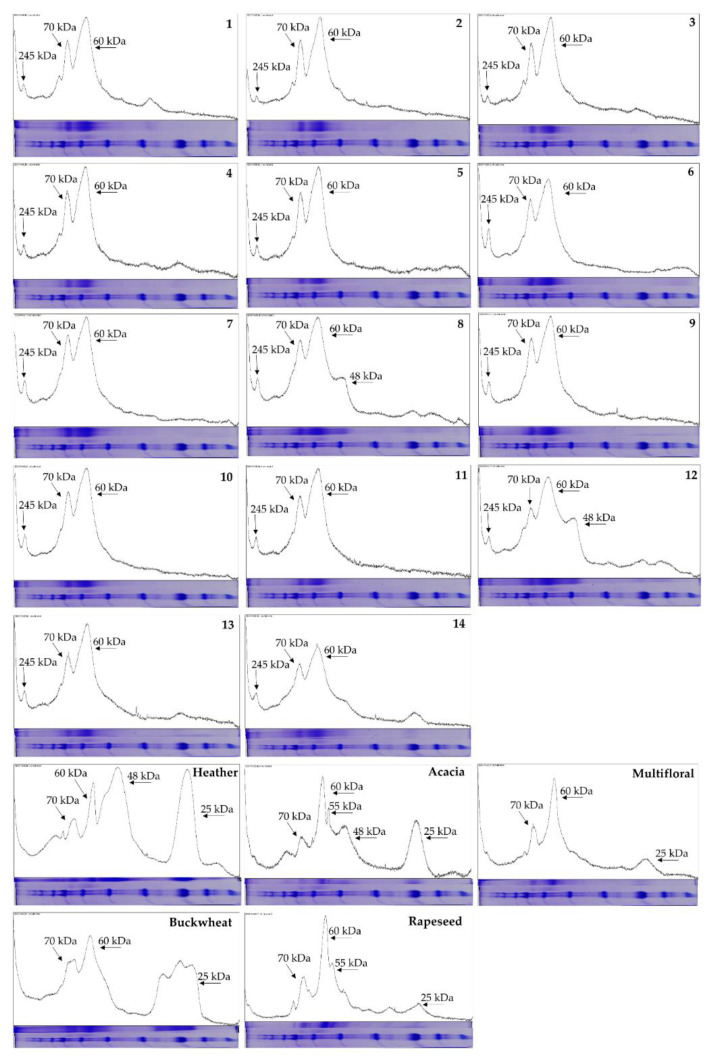
Detailed qualitative analysis of particular SDS-PAGE gel lanes obtained for honeydew honey samples (1–14) and nectar honeys in ImageJ software.

Detailed analysis of the protein profiles obtained with ImageJ software clearly indicates that the presence of a 245 kDa band is distinctive from honeydew honey. In all these samples, a repetitive band pattern on the gel is also visible: besides the already mentioned 245 kDa, there is also 80, 70, and a wide band of 60 kDa, which can be considered a specific fingerprint for honeydew honey. In two samples (8 and 12) additional 48 kDa and 25 kDa bands are also visible, but this result seems nonspecific and could be an effect of adulteration or nectar admixture (Figure 5). On the other hand, the presence of low-molecular-weight bands is characteristic for some nectar honeys: 55 kDa in acacia and rapeseed honey and 48 kDa in heather, acacia, and rapeseed honeys, and 25 kDa in all nectar honeys, probably of floral origin (Figure 5).

**Figure 6:**
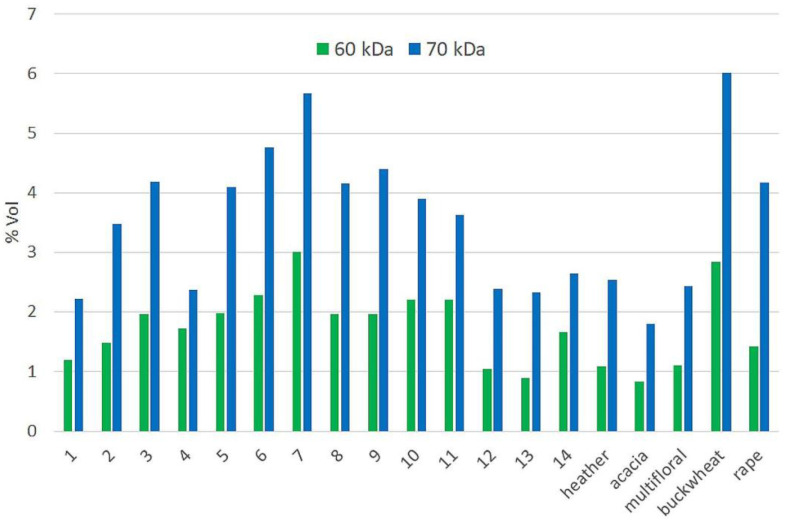
Quantitative analysis [%Vol parameter] of the two most intense bands on the CBB-G250 stained gel.

As the most intense bands on the gel obtained were those of 60 and 70 kDa, it was decided to perform a comparative quantitative analysis on a CBB-G250 stained gel using ImageMaster 2D software. This analysis did not provide conclusive results. Nevertheless, it can be clearly observed that the %Vol values obtained for the samples of honeydew honey with nectar admixture (1, 12, 13, 14) are similar to those of heather, acacia and multifloral honey. On the other hand, this parameter for pure honeydew honeys shows higher values similarly to buckwheat honey (Figure 6). The protein profile was previously successfully used for differentiation between honeydew honey and blossom honeys [63].

### 2.6. Statistical Analysis

**Figure 7:**
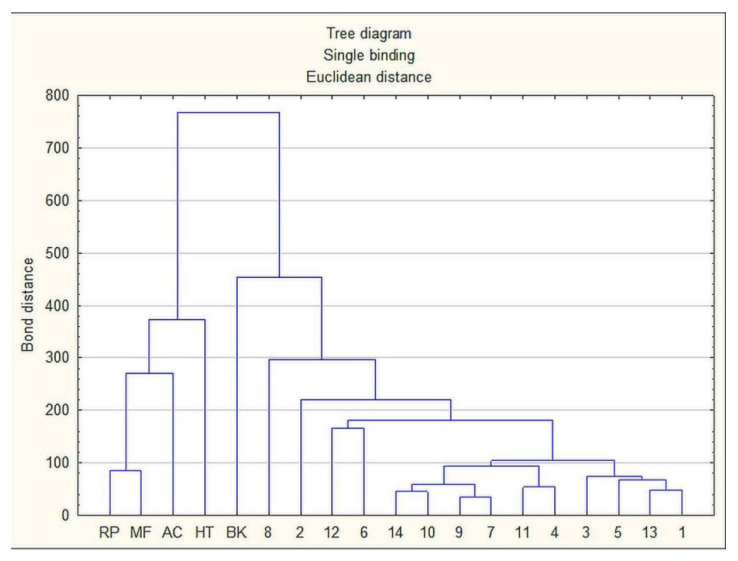
Tree diagram based on the average values of the physicochemical parameters and antioxidant activity between honeydew honeys and nectar honeys (complete linkage, Euclidean distance). (RP—rape, MF—multifloral, AC—acacia, HT—heather, BK—buckwheat).

Cluster analysis of physicochemical parameters and antioxidant activity calculated for all honey varieties tested showed the difference between honeydew honey and nectar honeys, which was confirmed by the highest bond distance (Figure 7). However, some similarities between buckwheat honey and honeydew honeys were found. Such analysis confirms that it is possible to detect the authenticity of honeydew honey based on physicochemical parameters but under the condition of analyzing many parameters, and even this will not give a definitive unequivocal result (similar to buckwheat honey). Thus, the proposed separation of polyphenols and sugars using the HPTLC technique, as well as electrophoretic separation, seems to be the fastest qualitative analysis allowing to distinguish honeydew honey from nectar honey.

The development of fingerprinting and profiling methods for the authentication of honeydew honey is currently a challenge in terms of specific properties of this type of honey. The occurrence of some specific phenolic compounds, minerals, sugars, and proteins in honeydew honey suggests possible beneficial health effects of this product; however, further research, especially in vivo, for proving this thesis is required.

## 3. Materials and Methods

### 3.1. Honey Samples

Fourteen samples of honeydew honey and five samples of other honey types (heather, acacia, buckwheat, multifloral and rape) collected directly from various beekeepers working in South-Eastern Poland in the 2019 beekeeping season were used. All samples were stored in the laboratory at 20 ± 2 °C until the time of analysis, and for no longer than 15 months. The botanical origin of honeydew honeys was declared by beekeepers, while other types were proved by melissopalynological analysis.

### 3.2. Physicochemical Properties

The water content was measured using the refractometric method, using a RHN1-ATC refractometer dedicated to honey (refraktometr.eu, Hradec Kralove, Czech Republic). Active acidity was determined in 20% aqueous honey solutions using the SevenCompact™ S210 pH-meter (Mettler Toledo, OH, USA) while total free acidity was determined by titration. A 50 mL of 20% honey solution was titrated with 0.1 M NaOH to reach a pH of 8.3 measured using the pH meter. The results were expressed in mval/kg. To determine the specific electrical conductivity, 20% honey solutions were used and the conductivity was measured using a conductometer CP-401 (Elmetron, Zabrze, Poland). The results (mS/cm) were calculated using a conductivity constant (K = 0.938 cm^−1^). All the above analyses were performed according to the procedures described in the Polish regulations for honey [64]. The color intensity was measured by colorimeter dedicated to honey color determination (Hanna HI 96785) and expressed in mm Pfund scale. All analyses were performed in triplicate.

### 3.3. Specific Optical Rotation

Specific optical rotation was determined according to IHC [65]. Briefly, 6 g of honey was dissolved in 20 mL of distilled water in a 100-mL volumetric flask. Then 5 mL of Carrez I solution (aqueous potassium hexacyanoferrate solution 10.6% *w*/*v*) was added and the mixture was shaken for 30 s. Next, 5 mL of Carrez II solution (aqueous zinc acetate 24% *w*/*v* and admixture with glacial acetic acid 3% *w*/*v*), was added, the mixture was shaken again for 30 s and made up with distilled water to 100-mL. After 24 h, the solution was filtered through filter paper and the filtrate was used to rinse and fill the polarimetric tube. The measurements of angular rotation were done at 20 °C in a manual polarimeter (POL-1, Optika Microscopes, Ponteranica, Italy). Finally, the specific rotation was calculated according to the following formula: SOR = α × 100/l × p, where α = angular rotation found; l = length in decimeters of the polarimeter tube and p = weight of honey.

### 3.4. Diastase Number Determination

Diastase number was determined by a spectrophotometric method with the Phadebas Honey Diastase test (Magle AB, Lund, Sweden) according to the manufacturer′s instructions. Five ml of a 1% honey solution in 0.1 M acetate buffer (pH 5.2) was heated for 5 min at 40 °C in a water bath. Then, a Phadebas Honey Diastase test tablet was added to each sample and after thorough mixing, the solution was incubated at 40 °C for 30 min. Then 1 mL of 0.5 M NaOH was added, the solution was mixed, filtered, and the absorbance of the filtrate was measured at 620 nm against blank (acetate buffer) using a Biomate 3 spectrophotometer (Thermo Scientific, Waltham, MA, USA). The values of the diastase number were calculated using the following formula included in the manufacturer’s instructions: DN = 28.2 × ΔA620 + 2.64.

### 3.5. Mineral Composition of Bioelements Using the ICP-OES Method

The assessment of selected minerals (Na, K, Ca, Mg, P, S, Fe, Mn, Zn, Cr, Cu, Sr, As) and toxic metals (Al, Cd, Pb) were determined by optical emission spectrometry with inductively coupled plasma (ICP-OES) using a Thermo iCAP 6500 spectrophotometer (Thermo Fisher Scientific Inc., Bartlesville, OK, USA) according to the procedure described by Dżugan et al. [39]. The detection limit for each element was determined at a level that was not lower than 1 µg/L. A curve fit factor for the elements studied was above 0.99. All analyses were performed in three independent replicates for each sample. Recovery determined by an internal standards addition method ranged from 93 to 105%. The method was validated using certified reference material (INCT-TL-1 tea leaves and NIES CRM No. 7 tea leaves). The response of the equipment was periodically checked with known standards. To identify the relevant measurement lines and avoid possible interferences, the method of adding an internal standard (yttrium and ytterbium) was applied.

### 3.6. Antioxidant Properties

#### 3.6.1. DPPH Test

The inhibition of DPPH (2,2-diphenyl-1-picrylhydrazyl) radicals was measured according to the method described by Dżugan et al. [15] with slight modifications. Twenty µL of each honey aqueous solution (20% *w*/*v*) was mixed with 180 µL of DPPH radical methanolic solution (0.1 mM) and kept in darkness for 30 min. In the control sample, the extract was replaced by water. After incubation, the absorbance of the test and control samples was measured against methanol at 517 nm in a microplate reader (EPOCH 2 microplate spectrophotometer, BioTek, Winooski, VT, USA). The results were calculated as % of inhibition and expressed as mg of Trolox equivalents (TE) per kg of honey (mg TE/kg) from the calibration curve prepared for Trolox in the range 25–300 nmol/mL (y= 15.543x, R^2^ = 0.998).

#### 3.6.2. ABTS Test

The inhibition of ABTS (2,2’-azino-bis(3-ethylbenzothiazoline-6-sulfonic acid)) radical cations was measured according to the method of Wilczyńska [44] with slight modifications. The ABTS^•+^ cation radical solution was prepared by dissolving 19.5 mg ABTS in 7.00 mL of distilled water which was mixed with 3.3 mg of potassium persulfate and stored in the dark for 24 h. The stock solution was diluted with 0.1 M phosphate buffer pH 7.4 to obtain an absorbance about 0.7 at 734 nm directly before analysis. Twenty µL of 20% *w*/*v* each honey aqueous solution was mixed with 180 µL of ABTS radical solution and kept in darkness for 6 min. In the control sample, the extract was replaced by water. After incubation, the absorbance of the test and control samples was measured against 0.1 M phosphate buffer at 734 nm in a microplate reader (EPOCH 2). The reduction of ABTS radical cations was calculated as % of inhibition of initial absorbance. The results were expressed as mg of Trolox equivalents (TE) per kg of honey (mg TE/kg) from the calibration curve prepared for Trolox in the range 5–60 nmol/mL (y = 9.0436x, R^2^ = 0.995).

#### 3.6.3. FRAP Test

The FRAP (Ferric Reducing Antioxidant Power) test was performed according to Dżugan et al. [15]. To 20 µL of the aqueous honey solution (20% *w*/*v*), 180 µL of the FRAP reagent (10 mM solution of 2,4,6-tripyridyltriazine (TPTZ) in 40 mM HCl, 20 mM aqueous FeCl_3_ solution, and 0.3 M acetate buffer pH 3.6 were mixed in the ratio 1:1:10) was added and after incubation at 37 °C for 10 min the absorbance of the reaction mixture was measured spectrophotometrically at 593 nm (EPOCH 2) against blank. The results were expressed as mg of Trolox equivalents (TE) per kg of honey (mg TE/kg) from the calibration curve prepared for Trolox in the range 25–300 nmol/mL (y = 0.155x, R^2^ = 0.999).

#### 3.6.4. Total Phenolic Content (TPC) Determination

The total content of phenolic compounds was determined using the Folin-Ciocalteu reagent according to Dżugan et al. [15] with minor modifications. To 20 µL of the aqueous honey solution (20% *w*/*v*), 100 µL of 10% Folin-Ciocalteu reagent was added followed by 80 µL of 7.5% (*w*/*v*) sodium carbonate solution. The samples were kept in darkness for 60 min and then the absorbance was measured against the blank at 760 nm using the microplate spectrophotometer (EPOCH 2). The total content of phenolic compounds was expressed in mg of gallic acid equivalents (GAE) per kg of honey (mg GAE/kg). The results were calculated based on a calibration curve prepared for gallic acid in the range 25–150 µg/mL (y = 0.341x, R^2^ = 0.995).

### 3.7. High-Performance Thin Layer Chromatography (HPTLC) Implementation

#### 3.7.1. Sugar Analysis

All standards and samples were applied as 8-mm bands within the distance of 8 mm from the lower edge of the HPTLC plate at rate of 50 nL/s using a semiautomated HPTLC application device (Linomat 5, CAMAG, Muttenz, Switzerland) according to the procedure described by Islam et al. [50]. The glucose, fructose, sucrose, maltose, melezitose, and raffinose standards were obtained by applying 2 µL, of the respective standard solutions (0.25 mg/mL) to determine their Rf values. For the analysis of sugars in the honey, 3 µL of each sample solution (1 mg/mL) were applied. Chromatographic separation was performed at ambient temperature on silica gel 60 F254 HPTLC plates (glass plates 20 × 10 cm), purchased from Merck (Darmstadt, Germany), in a saturated (33% relative humidity) automated development chamber (ADC2, CAMAG). The development chamber was saturated for 20 min, and the HPTLC plates were presaturated with the mobile phase (1-butanol: 2-propanol: aqueous boric acid (5 mg/mL) 30:50:10 *v/v/v*) for 5 min. The plates were automatically developed to 85 mm, and after drying for 5 min, they were analyzed under white light using an HPTLC imaging device (TLC Visualizer 2, CAMAG). The chromatographic images were digitally processed using specialized HPTLC software (vision CATS, CAMAG). After documentation of the initial chromatographic results, the HPTLC plates were derivatized with 2 mL of the aniline-diphenylamine-phosphoric acid reagent (CAMAG Derivatizer). After heating for 10 min at 115 °C and cooling to room temperature, the plates were re-analyzed under transmission white (T white) light using the HPTLC imaging device.

#### 3.7.2. Polyphenolic Profile Analysis

Analysis of polyphenolic profiles were performed on HPTLC Silica Gel 60 F254 plates (20 cm × 10 cm) purchased from Merck (Darmstadt, Germany). A 20 g aliquot of each honey sample was dissolved in 100 mL of acidified distilled water at room temperature. The solutions were then applied to a C-18 Sep Pack Cadrigde (Waters) conditioned with acidified water. Polyphenols were leached from the columns with methanol directly in a round bottom flask. A 5 µL of such prepared extract was applied to the plate as a 8 mm bands 11 mm from the lower edge, with a speed of 50 nL/s using a semi-automated HPTLC application device (Linomat 5, CAMAG, Muttenz, Switzerland). The chromatographic separation was performed in a chromatographic tank saturated for 20 min with the mobile phase composed of chloroform: ethyl acetate: formic acid (50:40:10 *v/v/v/v*), and developed to a distance 85 mm. The results obtained were documented using a HPTLC imaging device (TLC Visualizer, CAMAG) under white light, 254 and 366 nm. In addition, each plate was derivatized with p-anisaldehyde/sulfuric acid reagent using an automated derivatizer for TLC plates (CAMAG Derivatizer). After derivatization, the plates were heated at 110 °C for 10 min and imaged under white light and 366 nm. The obtained chromatographic images were analyzed using HPTLC software (Vision CATS, CAMAG).

#### 3.7.3. Protein Profiling by SDS-PAGE

One gram of raw honey was dissolved in 1 mL of deionized water containing 2% Nonidet P-40 Substitute, and 2% dithiothreitol. The samples were then mixed 2 to 1 with 4× concentrated standard Laemmli buffer and incubated for 5 min at 95 °C. After cooling, 30 µL of the samples were applied to 12% SDS-PAGE gels (with 5% stacking gels). Electrophoresis was carried out initially at 150 V (15 min) and then at 250 V for 2.5 h with cooling on an Omni PAGE WAVE Maxi apparatus (Cleaver Scientific Ltd., Rugby, Warwickshire, UK) according to the standard method of Laemmli [66], with the ROTI^®^Mark TRICOLOR ladder as a molecular weight marker. After electrophoresis, a gel for qualitative analysis was stained with Coomassie Brilliant Blue R, the second gel for quantitative analysis was stained with colloidal Coomassie Brilliant Blue G-250. The staining was performed overnight and then the gels were washed for 24 h with deionized water in order to remove the residue of the dye. Gels were scanned with Image Scanner III (GE Healthcare) and processed by LabScan 6.0 (GE Healthcare). The qualitative analysis of the gel was performed in ImageJ (1.52 a) software by generating graphs representing each lane on the gel. The quantitative analysis of the second gel was performed using ImageMaster 2D software. The parameter %Vol, which is the value of the band volume (resultant of area and intensity) normalized to the intensity of all the bands on the analyzed gel, was used to compare band intensities.

### 3.8. Statistical Analysis

The results are presented as mean values with standard deviations (SD). Significant differences (*p* < 0.05) between honey types were performed using Tukey’s test. The correlation between some parameters was calculated using Spearman’s correlation rank. To check whether honeydew honey can be distinguished from nectar honeys by physicochemical and antioxidant properties analysis, cluster analysis was performed. All calculations were obtained using Statistica 13.1 software (StatSoft, Inc., Tulsa, OK, USA).

## 4. Conclusions

Research on honeydew honey authenticity is connected to certain inherent difficulties compared to other varietal honey. Due to its origin from honeydew produced by plant-sucking insects, pollen analysis is not useful for discriminating this type of honey. Physicochemical parameters, included in legal standards, are mostly applied to confirm the market quality of honey but are insufficient in the case of deliberately adulterating honey. Based on the study of top-quality fir honeydew honeys produced in ecological region of Poland it was proposed that combining the HPTLC (sugar and polyphenols) and/or SDS-PAGE protein fingerprinting allows to accurately and precisely determine the origin of honeydew honey which is strongly expected by beekeeping industry to further secure honey quality.

## Figures and Tables

**Table 1:** The physicochemical parameters of honeydew and nectar honey expressed as mean ± SD, both included in legal requirements (water, pH, acidity, conductivity, HMF, and diastase number), and not legally required (color and specific rotation).

Honey Sample		Water Content [%]	pH	Acidity [mval/kg]	Conductivity [mS/cm]	HMF [mg/kg]	Diastase Numer [DN]	Colour [mm Pfund]	Specific Rotation [[α]D 20]
**Honeydew**	**1**	16.6 ± 0.4	4.91 ± 0.01	41.9 ± 1.9	1.234 ± 0.001	3.21 ± 0.27	14.03 ± 0.11	128 ± 1.4	+16.25 ± 0.08
	**2**	16.2 ± 0.1	4.88 ± 0.02	41 ± 0.6	1.414 ± 0.001	2.94 ± 0.03	16.26 ± 0.13	114 ± 2.1	+17.92 ± 0.25
	**3**	15.3 ± 0.3	4.78 ± 0.01	47.25 ± 0.25	1.392 ± 0.002	17.76 ± 0.35	15.13 ± 0.12	137 ± 0.7	+16.67 ± 0.17
	**4**	15.4 ± 0.3	5.04 ± 0.05	51.2 ± 0.3	1.333 ± 0.001	6.53 ± 0.05	13.46 ± 0.10	127 ± 2.8	+19.17 ± 0.17
	**5**	14.6 ± 0.3	5.07 ± 0.06	40.4 ± 1.0	1.262 ± 0.004	2.11 ± 0.14	14.00 ± 0.11	125 ± 0.7	+25.00 ± 0.33
	**6**	17.3 ± 0.1	4.56 ± 0.04	49.75 ± 0.68	1.176 ± 0.003	6.71 ± 0.01	15.55 ± 0.12	>150	+9.17 ± 0.08
	**7**	16.7 ± 0.3	4.38 ± 0.06	65.5 ± 1.3	1.053 ± 0.001	11.24 ± 0.10	15.55 ± 0.12	>150	+2.50 ± 0.25
	**8**	17.9 ± 0.0	4.52 ± 0.03	54.9 ± 1.7	1.302 ± 0.001	5.98 ± 0.18	13.24 ± 0.10	>150	+3.33 ± 0.17
	**9**	17.5 ± 0.2	4.52 ± 0.04	49.6 ± 1.4	1.169 ± 0.005	6.17 ± 0.07	11.55 ± 0.16	>150	+7.92 ± 0.25
	**10**	17.3 ± 0.1	4.50 ± 0.02	51.75 ± 0.55	1.204 ± 0.004	6.08 ± 0.01	11.12 ± 0.14	>150	+10.83 ± 0.08
	**11**	17.0 ± 0.1	4.85 ± 0.05	37.7 ± 0.3	1.267 ± 0.001	1.43 ± 0.26	10.02 ± 0.18	136 ± 1.4	+21.67 ± 0.08
	**12**	19.1 ± 0.1	4.40 ± 0.04	51.65 ± 0.35	1.181 ± 0.001	4.96 ± 0.10	15.01 ± 0.10	>150	+5.83 ± 0.17
	**13**	18.1 ± 0.1	4.36 ± 0.05	49.1 ± 0.3	1.087 ± 0.001	6.41 ± 0.84	11.01 ± 0.20	146 ± 1.4	+7.92 ± 0.08
	**14**	18.1 ± 0.1	4.47 ± 0.04	43.15 ± 0.65	1.028 ± 0.005	2.79 ± 0.01	8.92 ± 0.19	>150	+8.33 ± 0.08
	**min**	14.60	4.36	37.70	1.028	1.43	8.92	114	+2.50
	**max**	19.10	5.07	65.50	1.414	17.76	16.26	>150	+25.00
	**mean**	16.94	4.66	48.20 ^a^	1.222 ^a^	6.02 ^a,b^	13.20 ^b^	140 ^a^	+12.32 ^a^
	**SD**	1.24	0.25	7.19	0.118	4.22	2.31	12.3	7.05
**Heather**		16.60 ± 0.2	4.35 ± 0.05	31.20 ± 0.03 ^a,b^	0.771 ± 0.01 ^b^	0.94 ± 0.01 ^b^	28.25 ± 0.25 ^a^	57 ± 2.8 ^b^	−30.42 ± 0.18 ^b,c^
**Acacia**		17.95 ± 0.3	4.37 ± 0.01	6.95 ± 0.02 ^c^	0.355 ± 0.01 ^c^	4.50 ± 0.10 ^b^	15.16 ± 0.16 ^b,c^	10 ± 0.7 ^c^	−43.33 ± 0.42 ^b^
**Multifloral**		17.00 ± 0.2	4.55 ± 0.01	14.85 ± 0.03 ^b,c^	0.153 ± 0.01 ^c^	10.17 ± 0.07 ^a,b^	19.35 ± 0.14 ^b,c^	27 ± 2.1 ^c^	−19.58 ± 0.11 ^c^
**Buckwheat**		18.60 ± 0.2	3.99 ± 0.05	48.65 ± 0.04 ^a^	0.311 ± 0.01 ^c^	16.82 ± 0.03 ^a^	20.89 ± 0.13 ^c^	>150 ^a^	−17.92 ± 0.29 ^c^
**Rape**		18.90 ± 0.1	4.42 ± 0.01	18.50 ± 0.07 ^b,c^	0.208 ± 0.01 ^c^	1.60 ± 0.08 ^b^	18.20 ± 0.11 ^b,c^	39 ± 1.4 ^b,c^	−23.33 ± 0.33 ^c^

^a,b,c^—Means marked with different superscript letters within the column are significantly different (Tukey’s honest significant difference test, *p* < 0.05).

**Table 2:** Mineral content of honeydew and nectar honeys determined by the ICP-OES method. Mean values (mg/kg) of 3 independent replications are shown.

Honey Sample		Bioelements [mg/kg]											Toxic Metals [mg/kg]					Total [mg/kg]
		K	P	S	Mg	Ca	Na	Mn	Fe	Cu	Zn	Sr	Al	Ni	As	Pb	Cd	
**Honeydew**	**1**	2634	448.5	140.6	34.14	16.09	16.44	5.902	2.674	1.613	0.660	0.035	28.9	0.756	0.741	0.045	0.026	3331
	**2**	2889	546.1	98.7	40.74	30.68	8.19	5.602	1.815	1.856	0.867	0.068	17.3	1.412	0.766	0.027	0.037	3643
	**3**	2861	496.0	109.1	56.05	33.28	9.17	8.559	2.301	1.612	0.868	0.074	21.7	1.353	0.769	0.030	0.038	3602
	**4**	2951	526.3	141.1	36.13	12.87	9.93	6.222	2.692	1.871	0.805	0.035	31.8	1.097	0.780	0.034	0.030	3722
	**5**	2680	520.4	140.8	34.83	11.21	11.33	6.270	2.941	1.779	0.681	0.033	33.6	0.950	0.776	0.039	0.029	3445
	**6**	2386	496.2	134.4	65.10	38.77	11.81	7.898	2.370	1.680	1.882	0.096	27.1	0.978	0.776	0.050	0.041	3175
	**7**	2089	643.7	107.6	40.17	42.19	13.39	5.176	2.135	1.326	0.881	0.108	13.8	0.494	0.759	0.036	0.041	2960
	**8**	2813	538.0	103.6	38.22	82.07	14.04	5.282	4.659	1.552	1.009	0.127	17.8	1.381	0.763	0.085	0.027	3621
	**9**	2560	631.7	110.9	45.88	44.75	12.67	7.670	2.480	1.553	1.261	0.122	18.7	0.975	0.793	0.033	0.045	3439
	**10**	2461	633.3	105.7	45.22	45.15	12.18	7.500	3.142	1.530	1.249	0.123	18.1	0.958	0.746	0.028	0.046	3335
	**11**	2692	591.8	130.4	34.08	21.08	8.93	6.986	3.406	1.606	1.005	0.069	33.5	0.733	0.765	0.105	0.026	3526
	**12**	2494	444.7	81.9	33.53	115.78	13.81	5.470	1.880	1.170	1.227	0.154	13.0	0.810	0.753	0.017	0.019	3208
	**13**	2301	809.0	127.1	45.76	28.52	15.57	9.942	3.327	1.371	0.878	0.064	19.8	0.529	0.738	0.039	0.023	3363
	**14**	2207	452.5	107.9	36.36	31.27	10.65	9.046	7.075	1.816	9.184	0.065	24.8	0.988	0.781	0.120	0.035	2899
	**Min**	2088	444.7	81.9	33.53	11.21	8.19	5.18	1.81	1.17	0.66	0.03	13.00	0.49	0.74	0.02	0.02	2899
	**Max**	2950	809.0	141.1	65.10	115.78	16.44	9.94	7.08	1.87	9.18	0.15	33.57	1.41	0.79	0.12	0.05	3722
	**Mean**	2572	555.58	117.14	41.87	39.55	12.00	6.97	3.06	1.60	1.60	0.08	22.84	0.96	0.76	0.05	0.03	3376
	**SD**	259.5	97.21	18.01	9.07	27.60	2.47	1.47	1.38	0.20	2.15	0.04	6.92	0.28	0.03	0.03	0.01	249
	**%VC**	10.09	17.50	15.37	21.67	69.78	20.61	21.16	45.01	12.56	134.02	45.05	30.27	29.28	3.87	62.73	25.61	7.38
	**F-value**	168.8	5069	5524	144.6	2142	50.3	2254	32.2	440.9	316993	267.2	4327	8390	0.8	145.4	172.4	
	***p*** **-value**	<0.001													0.612	<0.001		

**Table 3:** Total phenolics content (TPC), reducing/antioxidant power (FRAP), and radical scavenging activity (DPPH and ABTS) of the analyzed honeys.

Honey Sample		TPC [mg GAE/kg]	FRAP [mg TE/kg]	DPPH [mg TE/kg]	ABTS [mg TE/kg]
**Honeydew**	**1**	834.08 ± 22.06	462.30 ± 11.52	249.69 ± 18.42	1606.96 ± 35.70
	**2**	635.42 ± 7.49	377.08 ± 15.74	228.80 ± 18.61	1367.68 ± 32.14
	**3**	737.35 ± 13.39	465.59 ± 14.38	256.46 ± 17.46	1538.11 ± 53.66
	**4**	853.42 ± 10.13	518.28 ± 15.82	262.28 ± 7.06	1693.43 ± 37.43
	**5**	769.35 ± 9.25	475.06 ± 10.11	255.87 ± 13.11	1600.24 ± 69.67
	**6**	1153.27 ± 25.31	609.67 ± 33.13	251.48 ± 21.59	1993.16 ± 66.97
	**7**	913.69 ± 13.96	523.22 ± 22.59	230.82 ± 19.28	1750.52 ± 29.45
	**8**	1289.43 ± 34.91	738.11 ± 37.22	280.57 ± 8.77	2222.37 ± 35.37
	**9**	942.71 ± 17.08	522.81 ± 19.46	238.30 ± 16.04	1748.85 ± 55.28
	**10**	988.10 ± 13.53	578.38 ± 31.48	242.45 ± 9.70	1782.43 ± 30.83
	**11**	860.12 ± 33.76	549.42 ± 30.19	263.47 ± 4.11	1653.97 ± 59.48
	**12**	990.33 ± 76.28	615.43 ± 30.31	260.86 ± 12.21	1959.58 ± 29.60
	**13**	844.49 ± 18.25	462.30 ± 28.28	209.92 ± 14.70	1593.52 ± 53.28
	**14**	948.66 ± 15.82	568.50 ± 14.93	258.84 ± 5.30	1779.07 ± 26.35
	**min**	635.42	377.08	209.92	1367.68
	**max**	1289.43	738.11	280.57	2222.37
	**mean**	911.46 ^a^	533.30 ^a^	249.27 ^a^	1734.99 ^a^
	**SD**	163.42	88.25	21.43	212.05
	**VC%**	17.93	16.55	8.60	12.22
	**F-value**	140.83	53.32	6.19	85.56
	***p*** **-value**	<0.001	<0.001	<0.001	<0.001
**Heather**		548.12 ± 10.10 ^c^	204.32 ± 7.64 ^b^	70.01 ± 4.72 ^c^	646.02 ± 6.63 ^c^
**Acacia**		110.37 ± 4.73 ^d^	32.25 ± 2.41 ^b^	15.66 ± 8.92 ^d^	141.72 ± 18.95 ^d^
**Multifloral**		249.01 ± 12.66 ^c,d^	128.58 ± 8.03 ^b^	42.84 ± 2.77 ^c,d^	443.19 ± 19.52 ^c,d^
**Buckwheat**		1574.40 ± 50.86 ^b^	644.39 ± 5.03 ^a^	311.01 ± 23.76 ^b^	2560.50 ± 26.99 ^b^
**Rape**		206.35 ± 6.67 ^c,d^	87.27 ± 4.53 ^b^	26.46 ± 6.74 ^c,d^	385.03 ± 18.69 ^c,d^

^a,b,c,d^—Means marked with different superscript letters within the column are significantly different (Tukey’s honest significant difference test, *p* < 0.05).

**Table 4:** Characteristic Rf values for detected phenolic compounds in tested honeys. Two specific bands for honeydew honey were highlighted in gray.

Rf	Colour		Honeydew (Band Present as a Percentage of All Samples)	Heather	Acacia	Multifloral	Buckwheat	Rape
	UV 366	VIS						
0.069	orange	grey	100%	+	+	+	+	+
0.121	blue	-	64%	-	-	+	-	+
0.265	pale orange	grey	93%	+	-	+	+	-
0.320	yellow	orange	43%	+	-	+	-	+
0.336	orange	-	36%	-	-	-	-	+
0.378	yellow	yellow	86%	+	-	+	+	+
0.486	orange	orange	71%	-	-	+	+	+
0.515	green/yellow	violet	57%	- (orange)	+	+	+	+
0.524	pink	-	43%	-	-	-	+	-
0.552	orange/yellow	-	64%	+	-	+	+	+
0.563	blue	-	79%	-	-	-	-	-
0.576	-	pink	100%	+	-	+	+	+
0.646	-	violet	100%	-	-	-	-	-

## Data Availability

The data presented in this study are available in the article.

## Sample Availability

Samples of the compounds are available from the authors for the limited time.
